# Prehabilitation: Impact on Postoperative Outcomes

**DOI:** 10.1097/AIA.0000000000000481

**Published:** 2025-05-05

**Authors:** Denny Z.H. Levett, Michael P.W. Grocott

**Affiliations:** Perioperative and Critical Care Theme, NIHR Southampton Biomedical Research Centre, University Hospital Southampton/University of Southampton, Southampton, UK

Major surgical interventions, particularly for intra-cavity cancer resections, impose significant physiological and psychological stress on patients. Postoperative complications are common, occurring in 15% to 40% of major (inpatient) surgeries, and are associated with prolonged hospital stays,^1^ increased health care costs,^2^ and reduced postoperative survival^3^ as well as delayed adjuvant therapy for cancer.^4^ The likelihood of experiencing postoperative complications is linked to several factors: the nature, extent, and duration of the surgical insult; the physiological and psychological resilience of the patient; and the care administered before, during, and after surgery (Fig. 1). It is increasingly clear that, given sufficient time and resources, interventions before surgery can augment both physical and psychological resilience.

**FIGURE 1:**
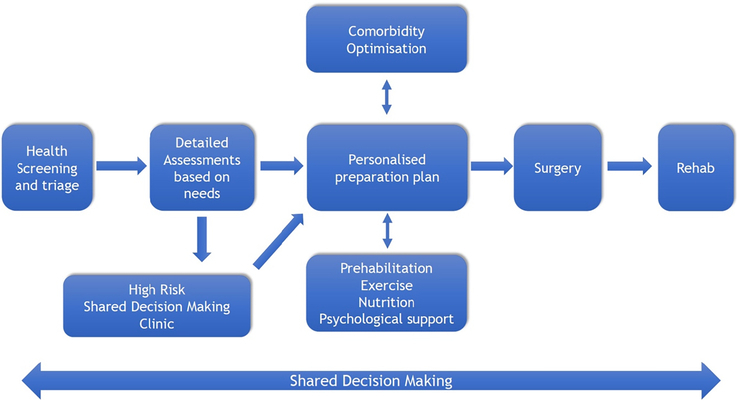
The perioperative pathway integrating prehabilitation, comorbidity optimization, and shared decision-making.

Prehabilitation is a multimodal intervention before surgery that aims to optimize functional capacity and physiological reserve to allow patients to withstand surgical stressors, improve postoperative outcomes, and facilitate recovery. Prehabilitation involves screening, needs-based assessment, and the individualized prescription of one or more of exercise, nutrition, and psychological interventions supported by behavior change techniques.^5^

The concept of prehabilitation has evolved from unimodal approaches focused principally on physical exercise training or nutrition to comprehensive multimodal strategies addressing multiple risk factors and supported by behavior change interventions.^6^ While all patients may benefit, to varying degrees, from prehabilitation interventions, individualizing therapy maximizes service efficiency and cost-effectiveness.^7^

Prehabilitation, as defined above, should be differentiated from the management of long-term conditions, commonly referred to as comorbidity/multimorbidity management, before surgery, which typically requires modification of pharmacological interventions under specialist guidance (eg, anemia, diabetes, COPD). Prehabilitation and comorbidity management are complementary, probably synergistic, and should occur in parallel, but are distinct interventions.

The notion of reframing “waiting lists” as “preparation lists” has highlighted the opportunities available when preoperative patient evaluation takes place early in the surgical journey, ideally as close as possible to the “moment of contemplation of surgery.” Early screening enables the identification of modifiable behavioral and comorbidity risk factors and preliminary triage of the patient’s prehabilitation needs and their perioperative risks. This, in turn, facilitates (i) shared decision-making; (ii) comorbidity management; and (iii) prehabilitation. Patients identified as at increased risk during screening undergo targeted assessments, which inform individualized domain-specific (eg, exercise, nutrition, psychology) prescription of needs-based interventions (Fig. 1).

This manuscript reviews the impact of prehabilitation on postoperative outcomes, including patient-centered, clinical, and cost-effectiveness measures, and the opportunities available for the implementation of prehabilitation into surgical pathways.

## DOMAINS OF PREHABILITATION

### Exercise

Physical activity is defined as any and all bodily movements that require skeletal muscle contraction and energy expenditure. Exercise is a subset of physical activity that involves specifically planned, structured, and repetitive movements that aim to improve performance or physical fitness.^8^ Aerobic exercise training relies on increased oxygen delivery to fuel continuous and moderate-to-high-intensity dynamic activities, which involve large muscle groups such as running and cycling. It increases heart rate and energy expenditure. Regular aerobic exercise training improves cardiovascular system and skeletal muscle function which leads to increased aerobic exercise capacity (VO_2_peak; anaerobic threshold) and endurance performance.^9^ Resistance exercise involves moving against a resistance with the aim of increasing muscular strength, power, bulk, or endurance. This is achieved by varying the resistance, the number of repetitions of the exercise in a set, the number of sets, and the rest between sets.^10^

Reduced cardiorespiratory fitness and functional capacity are associated with adverse surgical outcomes.^11,12^ Surgical stress triggers an inflammatory response leading to metabolic dysregulation, muscle catabolism, and impaired wound healing.^13^ Prehabilitation enhances physiological reserve and thereby potentially reduces the incidence of postoperative complications.^6,14^ Studies have shown that preoperative exercise programs can improve oxygen uptake (VO_2_ max),^15^ enhance muscular strength,^16^ and facilitate early mobilization postoperatively.^16^

Assessment of functional capacity using a validated test before the commencement of a prehabilitation program allows the exercise program to be tailored to the patient. Cardiopulmonary exercise testing is the gold standard for assessing functional capacity and may also be used to optimize comorbidities (arrhythmia control, bronchospasm) and to prescribe exercise.^17^ Alternative simpler functional tests include the shuttle walking test, the sit-to-stand test, the timed up and go test (TUG), and the 6-minute walk test, although these may be less sensitive to changes in fitness and may have ceiling effects in healthy individuals.^18^

Exercise programs should be based on the patient's baseline fitness and prescribed and reported using the FITT principle.^19^ The FITT principle specifies the frequency (how often?), intensity (how hard?), time (how long?), and type (aerobic vs. resistance; intervals vs. continuous) of exercise. The prescription should also include instructions for progressing exercise intensity as fitness improves to maintain the training effect.^10^ Patients should be instructed to estimate the intensity of exercise using perceived exertion (eg, 10-point Borg Scale), the talk test, and/or heart rate zones (Table 1).

**TABLE 1:** Methods to Evaluate Exercise Intensity

Evaluation method	Low intensity	Moderate intensity	Vigorous intensity
Borg Scale 0-10 rest—maximal	0-4 Rest-moderate	5-7 Somewhat hard-hard	8-10 Very hard-maximal
Talk test	Speak comfortably	Talk but can’t sing	Few words only
Breathing/speaking	• Easy to breathe • Can carry on a conversation	• Breathing more heavily • Can only complete 1-2 sentences	• Breathing very hard • Can only say a few words
Heart rate range	<55% maximum heart rate	55%-75% maximum heart rate	>75% maximum heart rate
Example exercise	Slow walk	• Brisk walk • Cycle <10 miles/h • General gardening • Dancing	• Running • Cycle >10 miles/h • Heavy gardening • Climbing stairs • Aerobic dancing

Unfortunately, the reporting of exercise interventions in prehabilitation trials is suboptimal. In a recent scoping review of exercise interventions in prehabilitation randomized controlled trials, only 44% provided sufficient details to allow replication of interventions, including how and when they were administered and progressed, and only 15% reported who delivered/supervised the exercise program.^20^ This makes it challenging to reproduce an exercise intervention, compare interventions, or implement an intervention across a health system. The biological effects of different exercise intensities and modalities are different: the anti-inflammatory/immune modulating effect of high-intensity exercise is dissimilar to low intensity walking, and consequently, their clinical effects may not be the same, for example, tumor regression. All exercise may not therefore be equal, and it is critical that the type and exercise intervention are accurately prescribed and reported. Furthermore, only 45% of prehabilitation trials reported adherence to the exercise intervention. Failure of an exercise intervention may be because of a lack of efficacy of the exercise itself, but it also could be due to a failure of adherence because of the method of implementation. Thus, it is important to report not only the details of an exercise intervention but also how it was implemented. Adherence to an exercise program may also be impacted by the degree of supervision (reported adherence rates vary from 16% to 97%). A recent systematic review reported that the efficacy of exercise prehabilitation was greater in programs with more than 1 supervised exercise session a week.^21^ Supervision may be of particular importance for high-risk patients or frail patients who may lack the confidence to exercise alone.

Early prehabilitation studies involved predominantly aerobic exercise interventions, given the reported increased risk of complications in patients with reduced aerobic exercise capacity.^15,22^ High-intensity interval programs (HIIT) were chosen primarily because interval programs improve fitness more quickly than moderate continuous exercise programs, time is limited in preoperative pathways, and they are more effective.^23^

Resistance exercise has increasingly been combined with aerobic exercise training in prehabilitation programs with the aims of improving muscle strength and mass, preventing sarcopenia, and ameliorating the effects of cancer cachexia.^24,25^ Combining aerobic and resistance exercise increases the intervention burden for patients, and further research is needed to evaluate the impact on surgical outcomes. There are no RCTs that compare aerobic versus resistance versus combined exercise. A recent systematic review of unimodal exercise prehabilitation concluded that prehabilitation reduced postoperative complications and length of stay. All 29 studies that included whole-body exercise included aerobic exercise; 13 studies included a combined program of aerobic exercise and resistance exercise.^26^ No studies evaluated resistance exercise alone. Further high-quality studies comparing resistance and aerobic exercise are needed to clarify the most effective type of exercise program for patients before surgery. Recent systematic reviews confirm that exercise interventions before surgery are acceptable to patients, feasible and safe.^14,26^

Increasing evidence suggests that exercise has antitumor and cancer preventive effects and that it may augment the efficacy of other neoadjuvant cancer treatments, including chemotherapy, radiotherapy, and immunotherapy.^27^ In colorectal cancer, tumor regression was increased 12-fold in patients participating in a supervised, personalized HIIT aerobic exercise program when compared with controls.^28^ This finding has subsequently been replicated with HIIT training in esophageal cancer^29^ and in prostate cancer.^30^ The mechanisms of action of exercise in prehabilitation have not been clarified, but proposed pathways include modulating tumor hypoxia, enhancing innate immunity, and modifying the inflammatory response.^31^ Further clinical trials are required to clarify the mechanism and explore the tumor regression effect of exercise in neoadjuvant therapies. Furthermore, there is an inverse dose-response relationship between exercise and long-term mortality after cancer treatment, emphasizing the importance of maintaining the changes in exercise behavior that prehabilitation programs initiate.^32^

Exercise interventions also have an important impact on mental health reducing both anxiety and depression which are prevalent in cancer surgery patients.^33^ This effect is mediated by exercise releasing endorphins and other neurotransmitters that enhance mood.^33^

In summary, exercise interventions before surgery improve surgical outcomes and enhance overall health and recovery and may improve quality of life. Further research should focus on the standardization of exercise interventions, defining the optimum type of exercise, exploring the mechanisms by which exercise impacts tumor regression, and exploring the effects of prehabilitation interventions on long-term exercise behavior and health outcomes.

### Nutrition

Malnutrition is defined as a state resulting from a lack of intake or uptake of nutrition that leads to altered body composition and body cell mass and reduced function.^34^ Malnutrition is an established risk factor for poor surgical outcomes, including increased postoperative morbidity and mortality, and extended length of hospital stay.^35,36^ Malnutrition also impacts the costs of care with 30% to 50% higher hospitalization costs being incurred by malnourished compared with well-nourished patients.^37^ Malnutrition is particularly common in cancer surgical patients, with a prevalence between 20% and 70%.^38^ Malnutrition in cancer may be caused by reduced food intake secondary to nutritional impact symptoms (reduced appetite, disturbed GI function, pain, difficulties chewing or swallowing), which in turn may be caused by the tumor or the adverse effects of cancer treatment. Many surgical patients are also sarcopenic, defined as reduced skeletal muscle mass in association with reduced muscle strength and function. Sarcopenia may occur as a consequence of aging and the frailty phenotype^39^ or secondary to cancer-related cytokine-mediated inflammation and cancer cachexia^40^ and is associated with adverse outcomes after surgery.

Nutritional prehabilitation aims to mitigate the impact of malnutrition. All surgical patients should be screened for malnutrition using a validated tool such as NRS-2002, Nutrition-Risk Screening Tool,^41^, PG-SGA, Patient-Generated Subjective Global Assessment,^42^ and MUST (Malnutrition Universal Screening Tool)^43^ (Table 2).^46^ Patients identified as at-risk should be formally assessed by a registered dietician or nutritionist using a validated nutritional assessment tool such as the Subjective Global Assessment (SGA), Patient-Generated-SGA (PG-SGA),^44^ or the GLIM.^45^ Body composition analysis using dual-energy x-ray absorptiometry (DXA), bio-electrical impedance analysis (BIA), CT, or MRI may be used to identify sarcopenia, although this remains primarily a research tool currently. Functional assessments include whole-body exercise tests and hand-grip strength using dynamometry. Dynanometry may be used to monitor response to interventions^47^ but its utility as a screening tool is less certain^48^ (Table 2).

**TABLE 2:** Nutritional Screening and Assessment

Nutritional screening	Nutritional assessment by dietician or nutritionist			
	Nutritional assessment tools	Physical assessment	Body composition	Functional assessment
Validated tools • PG-SGA short form^42^ • NRS-2002^41^ • MUST^43^	PG-SGA^44^ Subjective global assessment • Weight loss • Metabolic demand • Physical exam • Functional status	• Weight • Weight history • Anthropometry	• CT • MRI • Bioimpedance	• CPET • Hand grip • Shuttle walk • Timed up and go • 6-min walk test
Components: Weight loss • >10% loss in 6 mo • 5% loss in 3 mo BMI <18.5 Reduced intake • (<50% of normal intake in last 7 d)	GLIM^45^ • Reduced food intake • Weight loss • Reduced muscle mass • Reduced body mass			

Nutritional interventions should be targeted to the patients’ needs. Universal nutritional interventions include dietary counseling that includes advice on healthy eating and protein intake (aiming for 1.5 g/kg) and strategies to cope with nutritional impact symptoms such as nausea, reduced taste, and reduced appetite (Table 3). Targeted interventions including oral nutritional supplements (ONS) are recommended if requirements are not being met with food. ONS should be considered in all malnourished patients if the window before surgery is short.^49^ Adherence to advice may be facilitated by dietary counseling and follow-up and behavioral change support.^50^

**TABLE 3:** Nutritional Interventions, Indications, and Targets

Intervention	Indications
Dietary advice	• Aim for 25–30 kcal/kg per day • Aim for 1–1.5 g protein/kg (ideal body weight if BMI ≥30) per day • Food fortification and increased calorie density if low appetite • Individualized recommendations to address barriers to meeting nutritional requirements, for example, nausea.^45^
Oral nutritional supplements	• If unable to meet requirements above with food alone • Protein fortified to meet protein requirements
Immunonutrition	• ONS enriched with arginine, omega-3 fatty acids, and ribonucleotides
Probiotics, prebiotics, synbiotics	• No standardization of preparations, not routinely recommended, may reduce infective complications

Evidence supporting the use of immunonutrition before major cancer surgery is increasing. A recent metanalysis reported reduced postoperative morbidity and length of stay with oral nutritional supplements that are enriched with arginine, omega-3 fatty acids, and ribonucleotides irrespective of nutritional status.^51^ ESPEN guidelines recommend that malnourished cancer patients undergoing major surgery should receive 7 to 10 days of immunonutrition enriched ONS before surgery, although these supplements are not universally available in all health care systems.^49^ Probiotics are live microorganisms that confer beneficial effects to the host when given in sufficient quantities. Probiotics survive digestive processes during transit through the gastrointestinal tract, with the majority of their activity being in the colon.^52^ Prebiotics are food ingredients (such as dietary fiber) that are not digested in the upper gastrointestinal tract and stimulate the growth or activity of selective bacterial species in the colon.^53^ When prebiotics and probiotics are combined in a single preparation, they are known as synbiotics. Meta-analyses in major abdominal surgery suggest a reduction in postoperative infective complications with the use of probiotics, prebiotics, and synbiotics.^54^ However, a lack of standardization of preparations, duration of treatment, or route of administration means they are not routinely recommended currently.^49^

Specialist interventions include enteral nutrition and/or parenteral nutrition, which are recommended when a patient cannot meet their nutritional requirements through oral intake.^49^

A recent umbrella systematic review of randomized controlled trials of nutritional prehabilitation alone and in multimodal prehabilitation programs concluded that it is associated with a reduction in length of stay, postoperative complications, quality of life, quality of recovery, and mortality in major surgery.^14,55^ However, the quality of the primary studies was limited (small, single-center studies with heterogenous interventions), so high-quality multicenter trials with a standardized intervention are needed to delineate the size of this effect and the optimum intervention.

### Psychological and Behavioral Support

Preparing patients for surgery psychologically is an integral part of prehabilitation. Patients experience significant distress before surgery, and they may also experience mood disorders.^56,57^ This may be due to a recent cancer diagnosis or from being overwhelmed by the diagnostic process and the fear of surgery. Psychological distress increases the risk of mortality in cancer across a variety of tumor types and affects adherence to medical recommendations and postoperative recovery.^58^ Anxiety and depression have been linked to prolonged hospital stays, higher complication rates, and increased pain perception postoperatively.^59^ A growing body of evidence suggests that prehabilitation programs incorporating psychological support, including mindfulness-based interventions and coping strategies, can significantly reduce preoperative anxiety. Prehabilitation interventions incorporating cognitive-behavioral therapy and stress management techniques have shown promise in improving patient resilience.^57^

However, high-quality trials evaluating psychological interventions alone or in multimodal prehabilitation pathways are lacking.^59^ Psychological elements have been included in multicenter multimodal prehabilitation trials, but their core elements are often not well defined, which makes replication challenging.^6^ Future trials should focus on well-defined interventions and standardized outcome measures to identify the key elements of psychological interventions. A recent systematic review concluded that psychosocial interventions may impact clinical outcomes in multimodal prehabilitation trials, but with low certainty because of the quality of the primary trials.^14^

Patients should be proactively screened for psychological distress and mood disorders at the start of the surgical pathway using validated tools such as the hospital anxiety and depression scale,^60^ GAD 7,^61^ PHQ8,^62^ and distress thermometer.^63^ Patients with significant morbidity should be assessed by a health or clinical psychologist using a validated assessment tool such as CORE OM^64^ and triaged to an appropriate treatment pathway. For the majority of patients, psychosocial support can be provided by non-mental health specialist health care workers such as cancer nurse specialists, prehabilitation practitioners, exercise clinicians, or specialist nurses.^65^ A small minority may require formal counseling or psychological therapies with mental health professionals.

Prehabilitation is a behavioral intervention and patients need support to modify their behaviors which is challenging in the context of ill health and psychological distress.^59^ Behavior change techniques (BCT) (observable, replicable, and irreducible components of an intervention designed to alter or redirect causal processes that regulate behavior)^66^ can be used to influence health behaviors.^57^ BCTs relevant to prehabilitation include goal setting, action planning, self-monitoring, motivational interviewing, and social support (Table 4). These BCTs have been reported to improve adherence to prehabilitation programs, although the evidence is inconsistent,^67^ which reflects inconsistency in both definitions and the intervention itself.

**TABLE 4:** Behavior Change Techniques Used in Prehabilitation Programs and Their Impact

Behavior change technique	Impact
Goal setting^67^ • Realistic and tailored • Goal setting with • Action planning: Instruction of behavior Demonstration of behavior, Practice/rehearsals planning	Patients should set the goals: Specific • Achievable • Behavior focused Action planning: • When, where, how • Coping plans: to overcome barriers Associated with improved exercise adherence^67^
Self-monitoring^67^ • Wearable devices • Diaries • Activity logs Feedback • Regular contact • Support and encouragement • Motivational messages	Systematic tracking of behavior and progress improves adherence
Motivational interviewing^68^	To enhance and reinforce patients’ motivation to comply • Helps patients to internalize their reasons for prehab • Overcomes hesitations • Maintains engagement • Increases adherence
Social support^67^	Social support networks (family, friends, and fellow patients) can help patients cope with the physical and emotional challenges of cancer treatment and recovery, and improve long-term adherence to exercise advice

Behavioral change strategies are also crucial for long-term lifestyle modification, ensuring that the benefits of prehabilitation extend beyond surgery.^68^ Long-term maintenance of physical activity remains a challenge. Future research should focus on evaluating the impact of clearly defined BCTs on adherence and the role of digital technologies in providing continuous support and feedback.

## IMPLEMENTATION AND COST-EFFECTIVENESS

### Screening and Patient Selection

Understanding patient pathways to surgery and exploitation of opportunities for early screening, assessment, and intervention are fundamental to the effective delivery of prehabilitation, specifically, and perioperative care in general. Pathway mapping and modification are arguably the biggest enablers of improved perioperative care and prehabilitation.^69^

**FIGURE 2:**
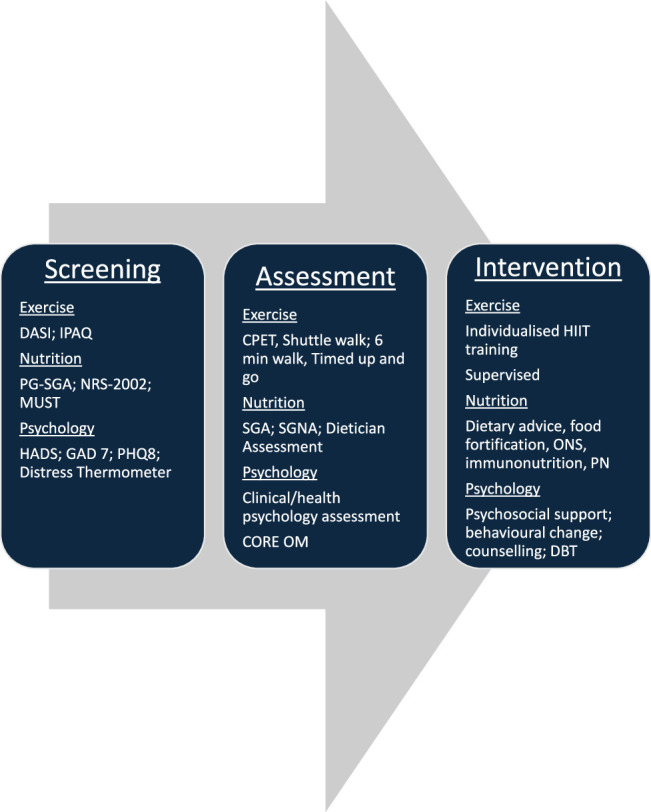
The prehabilitation pathway: screen, assess, intervene: needs-based care.

Screening all patients is essential and should occur at the earliest possible stage—ideally at the point of cancer diagnosis or initial surgical consultation—to allow adequate time for optimization before surgery (Fig. 1). Validated tools should be used to identify modifiable risk factors and comorbidities that may be optimized or impact perioperative risk (Fig. 2). Early screening permits a structured, stepwise approach to risk stratification that allows for personalized intervention planning, ensuring that resources are directed to those most likely to benefit.

Patients identified as high risk should undergo a comprehensive assessment based on need, which may include (CPET), psychological assessment, and nutritional assessment. A multidisciplinary approach, involving anesthesiologists, oncologists, physiotherapists, and dietitians, is crucial in determining individualized prehabilitation strategies.

### Personalized Intervention Plans

Prehabilitation interventions should be tailored based on individual assessments. The “universal-targeted-specialist” framework provides scalable implementation strategies.^7^ Universal interventions include general health advice and recommendations (eg, surgery schools),^70^ whereas targeted and specialist interventions involve individualized structured, supervised programs for higher-risk patients, likely to involve face-to-face or even hospital-based intervention for those at highest risk.

Personalization/individualization is key to maximizing adherence and outcomes. Factors such as baseline fitness level, comorbidities, treatment timelines, and psychosocial factors should be considered when designing intervention plans. Patients may have substantial needs meriting targeted or specialist intervention in 1 domain (eg, physical fitness), while not requiring support in other domains. Technological advancements, such as wearable fitness trackers and mobile applications, can facilitate real-time monitoring and engagement, enabling dynamic adjustments to intervention plans (see below).

While face-to-face supervised interventions have been shown to be much more effective than remote, patient-driven approaches (particularly for exercise), this approach is both costly and burdensome for patients in terms of travel and time. Community-based (eg, local gyms) may represent the best compromise with supervision possible by appropriately trained exercise professionals, convenience for patients, and benefits from additional health messaging achieved through face-to-face contact. Individualization to patient needs will determine the balance of risks and benefits between supervised/unsupervised and face-to-face or remote intervention. Guidance is needed on which patient groups require supervision for safety reasons, for example, aortic stenosis, poorly controlled atrial fibrillation, frailty, and falls risk.

### Integration Into Perioperative Pathways

A multidisciplinary approach is essential for the successful implementation of prehabilitation. Collaboration between anesthetists, surgeons, physiotherapists, dietitians, psychologists, and oncologists enhances patient outcomes.^71^ Embedding prehabilitation into routine perioperative pathways ensures accessibility and adherence.

Institutional adoption of prehabilitation requires the alignment of clinical workflows, patient education programs, and resource allocation. Standardized care pathways should integrate prehabilitation as a routine component of surgical preparation, with clearly defined referral criteria and intervention protocols (f1). Training programs for health care providers should emphasize the importance of prehabilitation and equip them with the skills to facilitate patient engagement, including motivational interviewing and behavioral change techniques.

### Technology Support for Prehabilitation

The application of digital technology to every stage of prehabilitation is appealing as a means to improve effectiveness and cost-effectiveness.^72^ Early screening is increasingly achieved through digital means (apps, web-based), with digitally naive patients supported by digital assistants to prevent digital exclusion. Digital support for interventions is increasingly advocated, and numerous apps and other resources have been developed. However, many of these digital interventions focus on promoting activity, rather than structured aerobic exercise, and there is substantial uncertainty about how effective they will be in improving outcomes when compared with current evidence-based interventions.^73^ Of particular concern is the observation that the frailest 20% of the population, at greatest risk of complications and thus with the greatest need for a prehabilitation intervention, may be the least likely to engage with a digital intervention.^74^ It is critical that studies of a digital intervention report the proportion of patients that did not engage with the intervention and their characteristics, as well as the experience and outcomes of those that do. Otherwise, we may unintentionally risk excluding the highest-risk patients where prehabilitation may have its greatest benefits.^75^

### Cost-effectiveness

Prehabilitation programs have demonstrated cost-effectiveness by reducing hospital length of stay, readmission rates, and the need for intensive care.^6,76^ Recent randomized clinical trials have shown that for every dollar invested in prehabilitation, significant cost savings are achieved through reduced complications and improved functional recovery.^77^ Health systems worldwide are increasingly recognizing the value of prehabilitation as an investment in preventive care, shifting resources toward preoperative optimization rather than reactive management of complications and focusing on the benefits to long-term health of improved diet and exercise.

## FUTURE DIRECTIONS AND RESEARCH PRIORITIES

Despite increasing evidence supporting prehabilitation, further research is needed to refine intervention protocols, enhance adherence strategies, and standardize outcome measures.^5^ The integration of digital health technologies, including remote monitoring and telehealth interventions, presents opportunities for increasing access to prehabilitation programs, but evidence of effectiveness is key. Improvement in screening approaches, including the development of a valid integrated screening tool for prehabilitation, would streamline the pathway to intervention. Similarly, identification of the most effective and cost-effective methods of assessment for each modality of prehabilitation will be an important research endeavor.

Future intervention research should focus on identifying the optimal duration and intensity of prehabilitation programs, as well as determining the most effective combination of interventions (eg, aerobic vs. strength training) for different patient populations. Large-scale multicenter trials are required to establish standardized guidelines and further validate the cost-effectiveness of prehabilitation. In addition, research should explore the long-term benefits of prehabilitation beyond the immediate postoperative period. Studies evaluating prehabilitation’s impact on long-term functional independence, quality of life, and overall survival will provide valuable insights into its broader implications for cancer survivorship.

Implementation science approaches should be utilized to address barriers to widespread adoption, including health care system constraints, patient engagement challenges, and provider training needs. Understanding the facilitators and challenges of integrating prehabilitation into standard care will help optimize its implementation and ensure sustainable delivery.

## CONCLUSIONS

Prehabilitation represents a transformative approach to perioperative care, emphasizing proactive patient optimization rather than reactive management of complications. By integrating exercise, nutrition, and psychological support, prehabilitation enhances surgical resilience, reduces complications, and accelerates recovery.

The evidence supporting prehabilitation continues to grow, highlighting its potential to improve patient outcomes and reduce health care costs. However, its full integration into routine clinical practice requires continued research, policy development, and health care system reorganization. Future efforts should prioritize standardization, accessibility, and cost-effectiveness to maximize the impact of prehabilitation in clinical practice.

Health care providers, policymakers, patients, and researchers must work collaboratively to ensure that prehabilitation becomes an integral part of perioperative care. With ongoing advancements in digital health, artificial intelligence, and personalized medicine, prehabilitation has the potential to revolutionize surgical preparation and recovery, ultimately improving patient outcomes and quality of life.
